# Australian Dentist's Knowledge and Perceptions of Factors Affecting Radiographic Interpretation

**DOI:** 10.1016/j.identj.2023.11.006

**Published:** 2024-01-06

**Authors:** Shwetha Hegde, Jinlong Gao, Rajesh Vasa, Shanika Nanayakkara, Stephen Cox

**Affiliations:** a Sydney Dental School, University of Sydney, Surry Hills, NSW, Australia; b Institute of Dental Research, Westmead Centre for Oral Health, University of Sydney, Westmead, NSW, Australia; c Applied Artificial Intelligence, Deakin University, Melbourne, Australia

**Keywords:** Cognition, Dental radiology, Interpretive errors, Perception

## Abstract

**Background:**

Errors of interpretation of radigraphic images, also known as interpretive errors, are a critical concern as they can have profound implications for clinical decision making. Different types of interpretive errors, including errors of omission and misdiagnosis, have been described in the literature. These errors can lead to unnecessary or harmful treat/or prolonged patient care. Understanding the nature and contributing factors of interpretive errors is important in developing solutions to minimise interpretive errors. By exploring the knowledge and perceptions of dental practitioners, this study aimed to shed light on the current understanding of interpretive errors in dentistry.

**Methods:**

An anonymised online questionnaire was sent to dental practitioners in New South Wales (NSW) between September 2020 and March 2022. A total of 80 valid responses were received and analysed. Descriptive statistics and bivariate analysis were used to analyse the data.

**Results:**

The study found that participants commonly reported interpretive errors as occurring 'occasionally', with errors of omission being the most frequently encountered type. Participants identified several factors that most likely contribute to interpretive errors, including reading a poor-quality image, lack of clinical experience and knowledge, and excessive workload. Additionally, general practitioners and specialists held different views regarding factors affecting interpretive errors.

**Conclusion:**

The survey results indicate that dental practitioners are aware of the common factors associated with interpretive errors. Errors of omission were identified as the most common type of error to occur in clinical practice. The findings suggest that interpretive errors result from a mental overload caused by factors associated with image quality, clinician-related, and image interpretation. Managing and identifying solutions to mitigate these factors are crucial for ensuring accurate and timely radiographic diagnoses. The findings of this study can serve as a foundation for future research and the development of targeted interventions to enhance the accuracy of radiographic interpretations in dentistry.

## Introduction

The interpretation of radiographs involves 2 identifiable and nonseparable components of diagnosis: identifying the abnormality on a radiograph (perception) and recognising the significance of the abnormal finding (cognition).^1^^,^^2^ These 2 components usually function effectively; however, they are not flawless, and errors can result from shortcomings in perception and cognition. Errors of interpretation, or interpretive errors, are a type of diagnostic error and have been described as incorrect interpretation^3^ or a discrepancy in the interpretation that significantly differs from the consensus among clinicians.^1^ Diagnostic errors in radiology can lead to misdiagnosis and inappropriate treatment, compromising safe and high-quality patient care. The consequences of diagnostic errors vary in severity and depend on the type of error, the stage of the diagnostic process, and the time taken to discover and rectify them. Errors may cause direct or indirect, permanent or temporary harm to the patient or may not result in any harm.^1^^,^^4^^,^^5^ Since Leo Garland's pioneering work in 1949,^6^ several studies have described the error types and their frequency in medical radiology.^3^^,^^7^^,^^8^ Medical radiology error rates have remained more or less unchanged since Garland's report.^9^ Currently, the global frequency of interpretive error ranges from 3% to 20%. The prevalence of interpretive errors can vary depending on the different regions worldwide, level of expertise and specific field within health care.^1^^,^^3^^,^^10^^,^^11^

Interpretive errors have been classified into 4 types based on the underlying processes: ^1^^,^^2^^,^^4^^,^^8^^,^^12^, ^13^, ^14^ near misses where the error is detected and corrected before the patient is harmed,^15^ delayed diagnoses where a diagnosis is not made within a reasonable amount of time,^16^ misdiagnoses where incorrect or wrong diagnosis occurs^16^ and errors of omission when the diagnosis or finding is missed on a radiograph.^16^ Factors contributing to interpretive errors in radiology are circumstances and actions that lead to or increase the risk of an interpretative error.^17^ Several factors contributing to interpretive errors in radiology have been identified in the literature, including clinical experience, clinical knowledge, clinician fatigue and burnout.^3^^,^^8^^,^^12^^,^^18^, ^19^, ^20^, ^21^ These factors are described under 4 domains, including image interpretation-related, image quality-related, clinician performance-related and patient-related factors in line with the WHO's International Classification of Patient Safety report.^17^ This classification has been used in the context of medical imaging,^22^^,^^23^ and surgical care in organ transplants.^24^ Image interpretation-related factors involve cognitive and perceptual processes, which can result in false positive and false negative diagnoses. Image-related factors pertain to aspects of image quality, including film quality and film faults. Clinician-related factors encompass factors such as lack of knowledge and clinical experience and ill health. Patient-related factors refer to situations such as patients experiencing pain, patients with dental fear and demanding patients.

Interpretive errors have been analysed using self-reported data, including surveys, clinical audits and autopsy results.^25^ Because of the dynamic nature of the diagnostic process, it is challenging to collect real-time data on interpretive errors except in experimental settings. In addition, data gathered in experimental settings cannot always be translated into real-life situations. In some cases where a definitive diagnosis is not possible based on a radiograph, 'errors' are just a difference of opinion rather than an incorrect diagnosis, and a clear-cut distinction between error and observer variation cannot be made.^3^^,^^26^ For these reasons, studying the frequency and underlying causes of errors can be challenging. Obtaining clinicians' perspectives through self-reported surveys that assess the knowledge and attitudes provides a snapshot of their understanding and perception of interpretative errors and highlights what is known and unknown in this area.^27^

While there is extensive data on this topic in the medical literature,^13^^,^^26^^,^^28^, ^29^, ^30^ there is limited understanding of how and why interpretive errors occur in dentistry. In addition, it is unclear whether dental professionals make the same mistakes or miss similar findings while analysing dental radiographs and whether the same factors influence their decision-making. Dentists routinely take and interpret radiographs, whereas, in a medical setting, radiographs are sent to a radiologist for interpretation and diagnosis. This distinction may also influence the incidence of errors of interpretation and its impact on patient care. A few studies have assessed the influence of clinical experience, stress and time pressure on the accuracy of radiographic diagnosis of certain common dental conditions.^31^, ^32^, ^33^, ^34^

Our recent systematic review identified factors affecting the interpretation of dental radiographs, including clinical experience, time pressure, clinical knowledge, case complexity and cognitive load.^35^ Understanding the causes and factors affecting interpretive errors will inform the development of solutions to minimise errors in clinical practice, improve diagnostic performance, and enhance patient outcomes. This survey aimed to evaluate dental practitioners' knowledge and perceptions about errors of interpretation of dental radiographs and the factors affecting them.

## Method

The study was approved by the Human Research Ethics Committee at the University of Sydney (2020/336). The survey was an online anonymised questionnaire, and informed consent was assumed when the participants completed and submitted the survey. Registered dentists and oral health therapists in NSW, Australia, were invited to participate in the survey. The survey was distributed via email and was also posted on social media and the ADA's e-newsletter.

The survey was carried out in 2 rounds between September 2020 and July 2021 and again between January 2022 and March 2022. The second round was conducted to boost the response rate. During this period, the data collected was monitored regularly for response patterns. A manual assessment was made to determine that data saturation was reached, as no new information was gathered with the new responses received similar to the data saturation approach^36^ used in qualitative research. Considering the voluntary nature of the survey, multiple survey platforms such as emails, social media and ADA's newsletter were used to improve coverage and response rate.

The online questionnaire was created using the Qualtrics platform (www.qualtrics.com), which was also used to distribute the survey to collect data. The questionnaire consisted of both closed and open-ended questions and was self-administered. Since no existing questionnaire in the dental and medical literature could be adapted to this study, the questions were based on the review of relevant literature on clinical decision-making, interpretive errors,^35^ and the clinical experience of the research team. Ten dentists were invited to participate in the pilot study to ensure the relevance and appropriateness of the survey questions. Two experts in survey methods and radiological diagnosis were also consulted to determine the content validity of the questionnaire. The pilot study and expert consultation helped refine and improve the quality of the survey questions and ensured that the final questionnaire was valid and reliable.

The questionnaire (Supplementary File Questionnaire) was organised into the following sections. The first part assessed the participants' awareness of errors in interpreting radiographs in their clinical practice. The second section explored the perception of the frequency of errors of interpretation. The third section explored the participants' opinions on the factors contributing to errors. The last part of the questionnaire collected participants’ demographic characteristics.

Data from the survey portal www.qualtrics.com was exported and analysed using IBM SPSS statistics software (Version 26, IBM SPSS Inc). Cronbach's alpha measured the internal consistency of the survey. Descriptive statistics were used to summarise and present the demographic characteristics of the participants and responses. Questions, where the respondents were required to rank the choices, were summarised using average weighted scores. Weights were applied in reverse, providing the highest score for the first choice and the lowest score for the last choice. The association between the categorical variables was assessed using the Chi-square test of independence (or Fisher's exact test when more than 20% of cells had expected frequencies <5). The Spearman rank correlation test assessed the correlation among the variables. The significance level for all statistical tests was set at *P*-value < .05.

## Results

One hundred eighteen dental practitioners responded to the survey. After removing the records with incomplete data, responses from 80 participants were included in the analysis (67.8%). Cronbach's alpha value for the questionnaire was 0.928, indicating a high internal consistency for the survey instrument.

### Demographic characteristics of participants

As presented in Table 1, the age of the participating dental practitioners ranged from 23 to 72 years, with 57.5% self-identified as females. The participants’ clinical experience ranged from 1 to 50 years, with most participants in the 10 to 19 years' experience range. As shown in Table 1, on average, the participants reported requesting radiographs for 6 patients per day, taking an average of 4 periapical radiographs, 5 bitewing radiographs and 3 orthopantomograms (OPGs) per day. The survey participants were employed in private practice, public practice, academia, or a combination of all three. (Table 1).

**Table 1:** Demographic characteristics of the study participants.

Characteristics	Value (*n* = 80)
Age (years)	
Mean (SD)	41.7±11.0
Minimum	23
Maximum	72
Gender n (%)	
Female	46 (57.5)
Male	22 (27.5)
Not specified	12 (15.0)
Type of clinical practice (choice count) n (%)	
Type of practice*	
General	35 (43.7)
Specialist	14 (17.5)
Not specified	31(38.8)
Sector†	
Private	21 (26.3)
Public	14 (17.5)
Both	11 (13.8)
Practice location†	
Metropolitan	13 (16.4)
Rural	5 (6.3)
Other (university academic)	2 (0.03)
Clinical experience in years	
Mean (SD)	17.1 (±11.8)
Minimum	1
Maximum	50
Time spent doing different activities per week (mean hours (SD))	
Clinical	27.90(±12.5)
Research	3.09 (±5.4)
Teaching	5.76 (±7.5)
Administration	4.83 (±5.9)
Patients per day requiring radiographs (mean ± SD)	6.2 (± 2.5)
Number of radiographs taken per day (mean± SD)	
Periapical	3.8 (±3.4)
Bitewings	4.9 (±3.7)
Panoramic radiographs (OPG)	2.7 (±2.7)
Other	0.49 (±1.04)
Imaging systems used in clinical practice n (%) (Choice count)†	
Photostimulable phosphor (PSP)	42 (52.5)
Direct digital (CCD, CMOS)	25 (31)
Analog films (chemically processed)	14 (17.5)
Other	20 (25)

⁎ The total number of responses does not add up to 100% for some variables due to missing responses.

† As the participants have selected more than one choice, the responses don't add up to 100%.

### Knowledge of the incidence and management of interpretive errors in clinics

Responses on how often interpretive errors occurred in their clinical practice were classified into 4 categories: regularly, occasionally, rarely and unknown, with the most common response category being ‘occasionally’. The majority of the participants (*n* = 49, 61.3%) indicated that interpretive errors were documented in their clinical practice. In addition, the majority of them (*n* = 61, 76.3%) indicated that their patients were informed about the error when it occurred. The survey found that a majority of the participants (*n* = 48, 60.8%) selected ‘sometimes’ to describe the extent to which errors were preventable. Interestingly, statistically significant (*P*-value = .02) differences were found in the responses of general practitioners (GP) and specialist dentists. More GPs (*n* = 12, 35%) thought that errors were preventable (always/most of the time) than specialists (*n* = 2, 14%).

### Types of interpretive errors and dental conditions frequently associated with interpretive errors

**Fig. 1:**
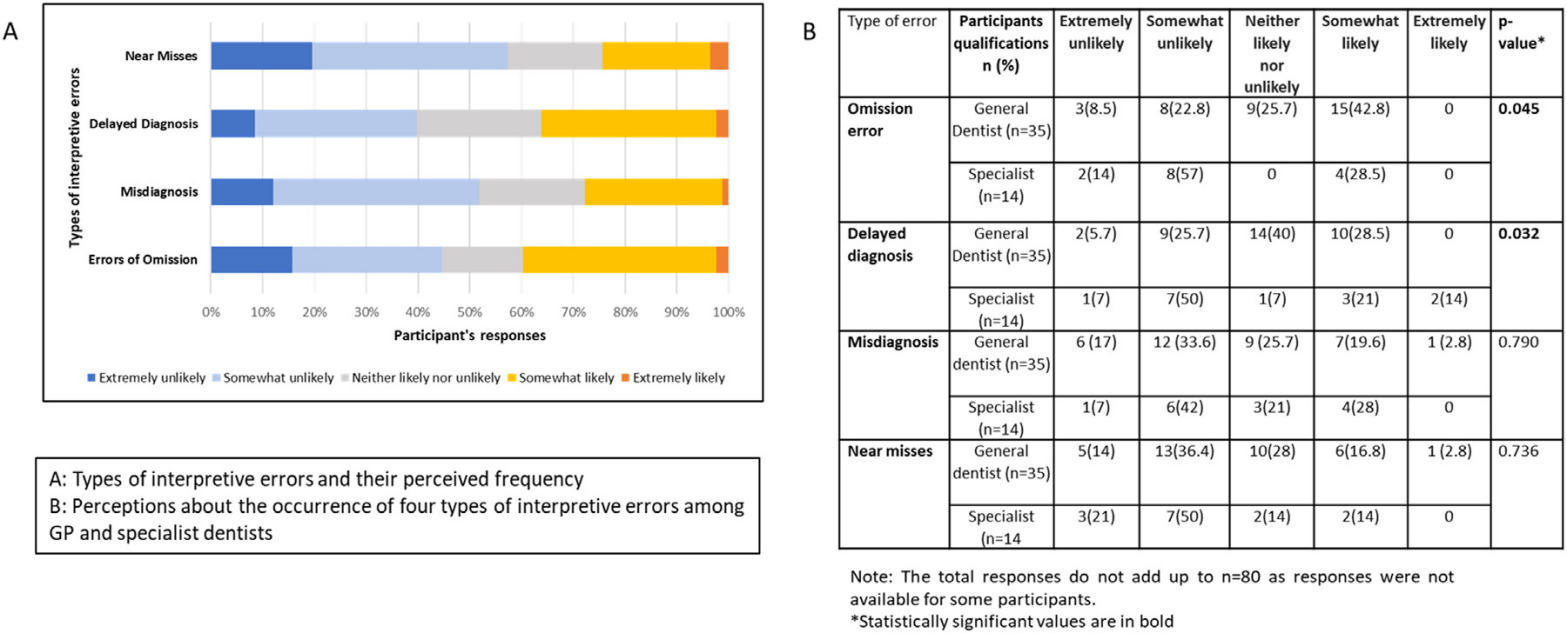
Composite figure showing the perceived frequency of interpretive errors and differences in the opinions of general practitioners (GPs) and specialists.

**Fig. 2:**
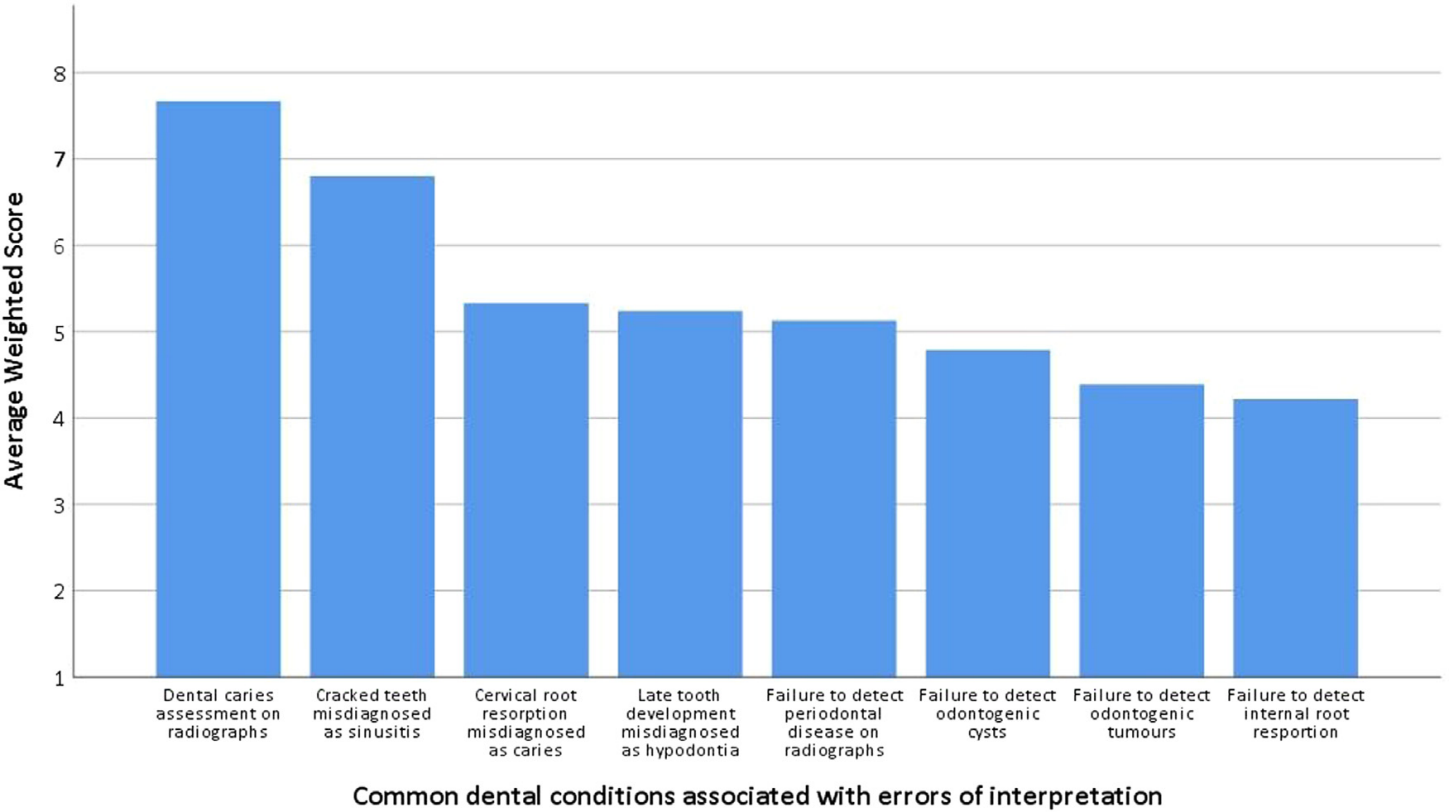
Rank scores of participants' perceptions of the likelihood of common oral conditions being incorrectly diagnosed on radiographs.

The participants acknowledged that all 4 types of errors could occur in clinical practice. As shown in Figure 1, errors of omission (*n* = 31, 39.2%) were identified as the most frequent type of error, followed by delayed diagnosis (*n* = 29, 36.7%), misdiagnosis (*n* = 22, 27.9%), and near-miss errors (*n* = 21, 26.6%). There were no statistically significant differences in the responses among different demographic categories, such as gender and location of practice, except in the responses given by general GPs and specialists. GPs suggested that omission errors occurred more frequently, while specialists suggested that delayed diagnosis occurred more frequently. In addition, the participants ranked dental caries, cracked teeth and cervical resorption of teeth as the most common dental conditions associated with interpretive errors (Figure 2).

### Factors contributing to interpretive errors

**Fig. 3:**
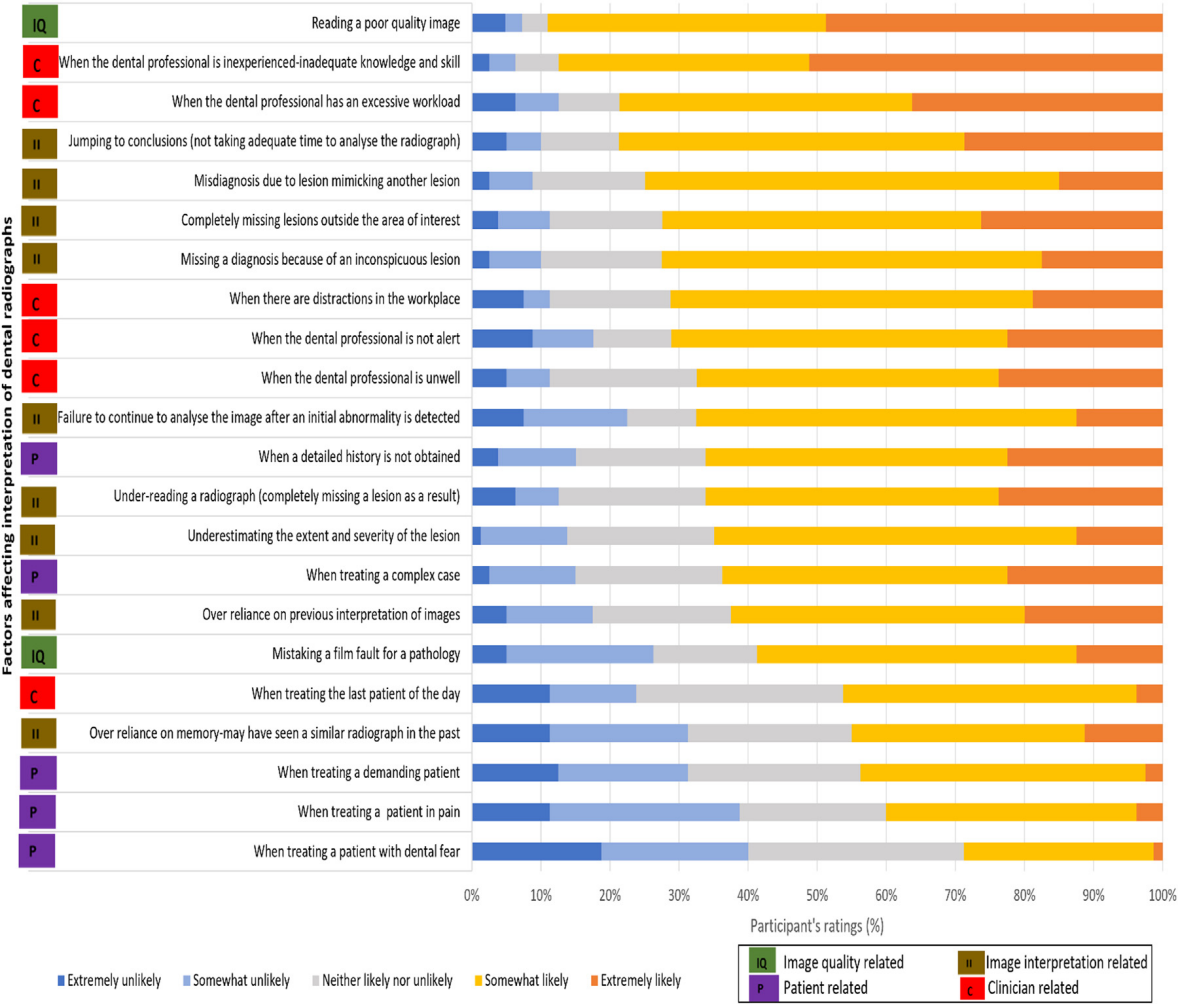
Participants' responses to the likelihood of factors affecting image interpretation. The factors have been listed in descending order from most likely to least likely as identified by the survey participants. The figure also uses a colour coding system to identify the factors affecting interpretive errors based on the 4 domains.

Twenty-two factors affecting interpretation errors are categorised into 4 domains, and the results are presented in Figure 3. The ratings are on a 5-point Likert scale ranging from extremely likely to extremely unlikely. The top 5 factors ranked most likely: poor image quality, lack of clinical experience, excessive workload, jumping to conclusions, and misdiagnosis due to a lesion mimicking another lesion, while the 5 least likely factors identified were treating the last patient of the day over-reliance on memory, treating a demanding patient, treating a patient in pain, and treating a patient with dental fear.

More GPs thought a lack of alertness and poor patient history could lead to interpretive errors (Table 2). In contrast, more specialists indicated that treating the last patient of the day and overly relying on memory could lead to interpretive errors. However, it is important to note that this result may be subject to bias since the sample size of specialists was small (*n* = 14). In addition, the survey found that more female respondents (*n* = 24, 52%) said that treating the last patient was extremely likely/somewhat likely to lead to interpretive errors than male respondents (*n* = 7, 31.8%) (Statistically significant; *P*-value = .046). However, there were no statistically significant differences in the responses of other demographic categories (GP vs specialists or dental practitioners in private and public dental practices).

**Table 2:** Differences in the responses between different participant groups regarding the likelihood of occurrence of factors affecting interpretation. Only statistically significant responses are shown.

	Participant's qualifications *N* (%)	Extremely unlikely	Somewhat unlikely	Neither likely nor unlikely	Somewhat likely	Extremely likely	*P*-value⁎
**Dentist not alert**	General Dentist (*n* = 35)	3 (8.5)	2 (5.7)	4 (11)	20 (57)	6 (17)	**0.025**
	Specialist (*n* = 14)	2 (14)	2 (14)	2 (14)	1 (7)	7 (50)	
**Treating the last patient of the day**	General Dentist (*n* = 35)	3 (8.5)	4 (11)	14 (40)	14 (40)	0	**0.039**
	Specialist (*n* = 14)	4 (28.5)	0	4 (28.5)	4 (28.5)	2 (14)	
**Poor patient history**	General Dentist (*n* = 35)	2 (5.7)	2 (5.7)	8 (22.8)	16 (45.7)	7 (20)	**0.046**
	Specialist (*n* = 14)	1 (7)	5 (35.7)	2 (14)	2 (14)	4 (28)	
**Over-reliance on memory**	General Dentist (*n* = 35)	4 (11)	8 (22.8)	12 (34)	10 (28.5)	1 (2.8)	**0.034**
	Specialist (*n* = 14)	1 (7)	2 (14)	1 (7)	6 (42.8)	4 (28.5)	

⁎ Statistically significant results.

The correlation coefficients between various factors that affect interpretation errors in radiographic images are presented in a table in the Supplementary Table S1. A significant positive correlation was found between over-reliance on previous interpretations and jumping to conclusions (*P*-value <.001). Several factors were found to have moderate positive correlations with the occurrence of interpretive errors, such as excessive workload, being the last patient of the day, workplace distraction, inexperienced dentists, patients with dental fear and absence of dental history (*P*-value <.001). Mistaking film fault for pathology had a moderate positive correlation (*P*-value <.001) with errors of interpretation, suggesting the significance of high-quality radiographs in diagnosis and the need for proper training and attention to technical aspects of radiography. On the other hand, a significant negative correlation was observed between clinical experience and perception of case complexity as a cause of the interpretive error (-0.258) (*P*-value <.05), indicating that more experienced clinicians were less likely to make errors when treating patients with complex dental problems.

## Discussion

The purpose of the survey was to evaluate the knowledge and perceptions of dental practitioners regarding errors in dental radiographic interpretations. The survey findings revealed that dental professionals considered errors of omission as the most likely type of error to occur compared to misdiagnosis, near misses and delayed diagnosis. In contrast, the medical literature suggests that delayed diagnosis and misdiagnosis are more likely to occur.^8^^,^^30^^,^^37^ In medicine and dentistry, errors of omission occur when a clinician fails to identify a pathology on a radiograph. They are more likely to occur in dental practice due to the setting where radiographic interpretation occurs, ie, in a busy dental clinic, compared to a dedicated image reading room in medical radiology. Hence, some radiographic findings could be missed. Additionally, there was a difference in the perceptions of GPs and dental specialists, where GPs believed that omission errors were more likely to occur, while specialists believed that delayed diagnosis occurred more frequently. This may be because specialists see more complex cases referred by GPs.

Dental caries, cracked teeth, and cervical resorption of teeth were identified as the most common dental conditions associated with interpretive errors, suggesting that these conditions pose particular challenges in radiographic diagnosis. Our findings are in agreement with several studies that have demonstrated that radiographic assessment of caries depth and cracked teeth were more likely to be misdiagnosed.^38^, ^39^, ^40^, ^41^, ^42^ The skills to detect and diagnose caries and cracked teeth develop as clinicians gain more experience. Moreover, the survey responses also showed that interpretation errors were less likely to occur with diseases such as odontogenic cysts and tumours. These conditions are encountered less frequently, so dental practitioners are likely to be more cautious and attentive during the diagnostic process.

The practice of dentistry requires a high level of attention and places a high demand on perception and cognitive functions. Factors such as the clinician's physical health and alertness, time of day, and workplace distractions, have been shown to influence cognitive and perceptual functions.^43^^,^^44^ Studies have shown that physical and mental fatigue can lead to interpretive errors among radiologists and are a well-known cause of such errors in medical radiology.^45^ In this survey, when the factors contributing to interpretive errors were ranked in the order of likelihood, the image quality-related, clinician-related, and image interpretation-related were prominent within the 50th percentile. In contrast, the patient-related factors dominated the 50th to 100th percentiles. Poor radiographic image quality can significantly hinder radiographic diagnosis by making it difficult or impossible for clinicians to visualise and accurately interpret the relevant anatomical structures or lesions. In addition, the participants also recognised the significance of clinician-related and image-interpretation-related factors in the occurrence of errors of interpretation. Our previous systematic review^35^ has identified that these factors lead to mental overload resulting in interpretive errors. Mental overload can be managed with cognitive aids such as checklists and machine learning supporting clinical decision support, as demonstrated in the medical radiology literature.^46^, ^47^, ^48^, ^49^

It is interesting to note that the participants did not perceive patient-related factors as significantly impacting the frequency of interpretive errors. This observation suggests that dental practitioners do not view these factors as significant contributors to interpretive errors. The influence of a patient's physical and mental state on a clinician's decision-making has been studied, revealing that patient behaviour, motivation for self-care, the patient-clinician relationship and the patient's mental health can affect the clinical decisions made by clinicians.^44^^,^^50^^,^^51^

### Limitations

This study had a limited sample size, with the majority of the participants practising in urban locations, limiting the generalisability of the results. Similar small sample sizes were observed in other survey-based studies conducted in Australian dental communities in the recent past.^52^, ^53^, ^54^ Furthermore, the voluntary nature of the survey could also result in selection bias. Although the open invitation to the survey was sent out to all dental practitioners, responses were not received from other dental practitioners, such as oral health therapists, who are also involved in the dental radiography and interpretation process. However, the survey population was representative when compared to the wider dental community using the most recent registrant data (Dental Board of Australia publication June 2023^55^). In addition, the inclusion of a proportionate number of specialists, especially oral radiologists, would have been beneficial in obtaining a more comprehensive understanding of factors affecting interpretive errors.

## Conclusion

The survey on the knowledge and perceptions regarding interpretive errors in dentistry provided valuable insights into the prevalence and factors affecting interpretive errors. The findings emphasised the need for careful attention to detail and continuous education in radiographic interpretation, especially in identifying errors of omission, which were found to be the most common. The study also highlighted the importance of various factors related to image quality, clinicians, and image interpretation which may contribute to interpretive errors. To reduce interpretive errors, the use of radiographic diagnostic aids, such as computerised decision support systems and machine learning algorithms and identifying potential solutions to minimise cognitive load should be considered. Future studies in collaboration with dental organisations should focus on gaining a deeper understanding of the pattern of image analysis by dental practitioners and identifying new insights into factors influencing their occurrence. Qualitative studies including focus group interviews, may also help in an in-depth understanding of the attitudes and perceptions of dental practitioners regarding interpretive errors.

## Conflict of interest

The authors declare that they have no known competing financial interests or personal relationships that could have appeared to influence the work reported in this paper.

## Appendix Supplementary materials

Image, application 1

## Footnotes

Supplementary material associated with this article can be found in the online version at doi:10.1016/j.identj.2023.11.006.
