# Supplementary material for: Australian Dentist's Knowledge and Perceptions of Factors Affecting Radiographic Interpretation
Source: Int Dent J. 2024 Jan 6;74(3):589–96. doi: 10.1016/j.identj.2023.11.006 (PMC11123563; doi:10.1016/j.identj.2023.11.006)
Supplement: Supplementary file 1 [file mmc1.pdf]

Supplementary 1: Survey Questionnaire

Dentist's Perceptions of Errors of Interpretation of Radiographs

Introduction and Survey information

<u>**Summary of the research project:**</u>

Errors in the interpretation of medical imaging are the most common causes leading to diagnostic error. In clinical dentistry, the rate and cause of error in interpretation of dental radiographs is not as well documented and studied. This survey aims to explore your perceptions of errors of interpretation on dental radiographs, causes and implications of these errors and strategies to minimise them. The participant information statement attached here describes the study in further detail.

This survey will take approximately 20 minutes to complete.

[Participant Information Statement]

Thank you for taking the time to participate in the survey!

Section 1

How important are **radiographs** in treatment planning for a patient?

Not at all important	Slightly important	Moderately important	Very important	Extremely important
○	○	○	○	○

How often do you encounter errors of radiology interpretation in your practice?

[ ]

When an error of radiology interpretation occurs, is it documented?

- ◯ Yes
- ◯ No

When an error in radiology interpretation occurs, is the patient informed?

- ◯ Yes
- ◯ No

To what extent do you feel that errors of radiology interpretation are preventable?

Never	Sometimes	About half the time	Most of the time	Always
◯	◯	◯	◯	◯

Section 2: Frequency of errors

Please **rate in the order of likelihood**, type of error likely to occur in your practice?

	Extremely unlikely	Somewhat unlikely	Neither likely nor unlikely	Somewha likely
1. **Error of Omission**: when no diagnosis is made	◯	◯	◯	◯
2. **Misdiagnosis**: when another diagnosis is made before the correct one	◯	◯	◯	◯
3. **Delayed Diagnosis**: when there is an unintentional delay in diagnosis	◯	◯	◯	◯
4. **Near Misses**: although an incident occurred that could have potentially lead to an adverse event if no harm came to the patient	◯	◯	◯	◯

Please rank the **conditions** where errors of interpretation on radiographs are likely to occur;

dental caries assessment of depth

cracked tooth misdiagnosed as sinusitis/TMD/atypical facial pain

misdiagnosis of late tooth development as hypodontia

failure to detect radiographic signs of periodontal disease

failure to detect radiographic signs of an odontogenic cyst

failure to detect radiographic signs of benign odontogenic tumour

cervical external root resorption misdiagnosed as root caries

failure to diagnose internal root resorption

any other cause : ________

Section 4: Contributing factors

Please rate the following **potential causes** for errors of radiology interpretation:

	Extremely unlikely	Somewhat unlikely	Neither likely nor unlikely	Somewhat likely
1. When the dentist is not alert	◯	◯	◯	◯
2. When the dentist has an excessive workload	◯	◯	◯	◯
3. When treating the last patient of the day	◯	◯	◯	◯
4. When the dentist is unwell	◯	◯	◯	◯

	Extremely unlikely	Somewhat unlikely	Neither likely nor unlikely	Somewhat likely
5. When the dentist is inexperienced-inadequate knowledge and skill	○	○	○	○
6. When a detailed history is not obtained	○	○	○	○
7. When treating a complex case	○	○	○	○
8. When treating a patient with dental fear	○	○	○	○
9. When treating a demanding patient	○	○	○	○
10. When treating a patient in pain	○	○	○	○
11. When there are distractions in the workplace	○	○	○	○

In your opinion, **how likely are the following to cause** errors of interpretation of radiographs ?

	Extremely unlikely	Somewhat unlikely	Neither likely nor unlikely	Somewha likely
1. Reading a poor quality image	○	○	○	○
2. Mistaking a film fault for a pathology	○	○	○	○
3. Jumping to conclusions (not taking adequate time to analyse the radiograph)	○	○	○	○
4. Under-reading a radiograph(completely missing a lesion as a result)	○	○	○	○
5.Over reliance on previous interpretation of images	○	○	○	○
6. Over reliance on memory-may have seen a similar radiograph in the past	○	○	○	○
7. Failure to continue to analyse the image after an initial abnormality is detected	○	○	○	○
8. Completely missing lesions outside the area of interest	○	○	○	○

	Extremely unlikely	Somewhat unlikely	Neither likely nor unlikely	Somewha likely
9. Misdiagnosis due to lesion mimicking another lesion	◯	◯	◯	◯
10. Underestimating the extent and severity of the lesion	◯	◯	◯	◯
11. Missing a diagnosis because of an inconspicuous lesion	◯	◯	◯	◯

Section 5: costs and consequences of errors of interpretations

In terms of **consequences of errors of interpretation to the patient**, please rate the likelihood of occurrence of following

	Extremely unlikely	Somewhat unlikely	Neither likely nor unlikely	Somewhat likely	Extremely likely
1. Severe harm or mortality	◯	◯	◯	◯	◯
2. Over treatment	◯	◯	◯	◯	◯
3. Under treatment	◯	◯	◯	◯	◯
4. Negatively affecting patient's quality of life-physical, mental	◯	◯	◯	◯	◯
5. Additional financial cost to the patient	◯	◯	◯	◯	◯

In terms of **consequences of errors of interpretation to the dentist**, please rate the likelihood of occurrence of following

	Not at all important	Slightly important	Moderately important	Very important	Extremely important

	Not at all important	Slightly important	Moderately important	Very important	Extremely important
1. Additional cost to the dentist to retreat the patient	○	○	○	○	○
2. Loss of business/client	○	○	○	○	○
3. Loss of reputation	○	○	○	○	○
4. Legal implications	○	○	○	○	○
5. Increase in the risk profile-insurance premiums	○	○	○	○	○

SECTION 5: Strategies to reduce errors in interpretation of radiographs

Please **rate the following activities in the order of their significance in reducing errors of interpretation** in the context of each patient

	Not at all important	Slightly important	Moderately important	Very important	Extreme importar
1. Seeking and analysing a patient's previous radiographs	○	○	○	○	○
2. Comparing a patient's current(latest) radiograph to their previous radiographs	○	○	○	○	○
3. Ensuring high quality images are used for diagnosis	○	○	○	○	○
4. Prescribing appropriate radiographs based on the patient's complaint	○	○	○	○	○

Please **rate the significance** of the following in **reducing errors of interpretation** of dental radiographs

	Not at all important	Slightly important	Moderately important	Very importa
1. Using a checklist or a template for analysis of radiographic images to standardise the process	○	○	○	○
2. Having reflective practice to critically analyse clinical decisions	○	○	○	○
3. Phone consultation with an expert	○	○	○	○
4. Machine learning systems that detect errors of interpretation	○	○	○	○
5. Machine-learning systems to facilitate diagnostic feedback-automated decision support	○	○	○	○
6. Further education and training to enhance diagnostic skills	○	○	○	○
7. Discussion of cases with colleagues	○	○	○	○

SECTION 6 : Participant Demographics

Age

Gender

Type of clinical practice : select all that is applicable

☐ general	☐ public	☐ metropolitan	☐ none
☐ specialist	☐ private	☐ rural	☐

other

Please indicate **number of hours spent per week** doing the following activities

- [ ] clinical
- [ ] research
- [ ] teaching
- [ ] administration

Please indicate the number of **years of clinical experience**?

Please indicate average number of your patients requiring radiographs per day?

Please select the types of radiographs frequently taken in your practice **and** indicate the number of each radiograph taken per day

- [ ] periapical radiographs
- [ ] bitewings
- [ ] panoramic radiographs(OPG)
- [ ] other

Type of imaging system used for intraoral radiography in your practice

Photo stimulable direct digital chemically other

phosphor (PSP)	(CCD/CMOS)	processed/wet films	
☐	☐	☐	☐

Phase 2 of this study involves focus group interviews to gain in depth understanding of the decision making processes involved in diagnoses on dental radiographs. It will also involve participation in a short radiographic diagnosis activity.
Would like to participate in the second phase of this study?

- ◯ Yes
- ◯ No
- ◯ ______ If yes, please enter your email address

Would you like to receive future communication with the results of the study?

- ☐ Yes
- ☐ No
- ☐ ______ If yes, please enter email address

Powered by Qualtrics

Table S1

Correlation among the various factors affecting interpretative errors and the frequency of interpretive errors. Correlation coefficient values are presented in this table.

		Clinician performance related factors			Patient-related factors		Image interpretation factors				Image quality-related factor
	Factors affecting errors of interpretation	Excessive workload	Dentist not alert	Dentist unwell	Complex case	Patients with dental fear	Under reading a radiograph	Jumping to conclusions	Satisfaction of search	Over-reliance on previous radiographs	Mistaking film fault for pathology
Demographic	Clinical experience				-0.258 *						
Clinician performance related factors	Excessive workload		0.562 **	0.594 **							
	Last patient of the day	0.488 **		0.571 **							
	Workplace distraction	0.517 **		0.515 **							
	Inexperienced dentist	0.446 **		0.426 **							
Patient related factors	Complex case					0.509 **					
	Demanding patient					0.583 **					
	When dental history is not obtained				0.418 **						
Image interpretation-related factors	Jumping to conclusions						0.588 **		0.498 **	0.707 **	0.474 **
	Over-reliance on memory (seen on another radiograph)							0.557 **	0.655 **	0.582 **	
	Over-reliance on previous radiographic interpretation						0.582 **		0.587 **		0.451 **
	Satisfaction of search						0.514 **				
	Missing a lesion outside the area of interest						0.478 **	0.508 **	0.569 **		0.408 **
	Misdiagnosis due to lesion mimicking other lesions										0.415 **

* Indicates p- value <0.05; **indicates p-value<0.001
